# Difficulty of isolating recalcitrant patients with infectious TB

**DOI:** 10.5588/pha.24.0048

**Published:** 2025-06-04

**Authors:** Y. Nagata, T. Zama, S. Hirao, M. Urakawa, M. Ota

**Affiliations:** Research Institute of Tuberculosis, Japan Anti-Tuberculosis Association, Matsuyama 3-1-24, Kekkaku, Kenkyusho, Kiyose, Tokyo, Japan.

**Keywords:** epidemiology, tuberculosis, Japan

## Abstract

**SETTING:**

Hospitals in Japan with more than five TB isolation beds.

**OBJECTIVES:**

Approximately 4,000 cases of sputum smear-positive TB were reported in 2022, who were isolated in a TB ward at least until they became sputum smear-negative. However, some recalcitrant patients are difficult to isolate because of their behaviour. This study aims to characterise recalcitrant patients with TB and determine why they left the hospitals.

**DESIGN:**

This was a descriptive study. We sent a self-administered questionnaire to the hospitals asking about recalcitrant inpatients with TB from April 2022 through March 2023.

**RESULTS:**

A total of 12 recalcitrant patients were identified, of whom 8 (66.7%) self-discharged, and the other 4 (33.3%) were discharged by the hospital. All were male with an average age of 58.9 years. The main reason why the patients were considered recalcitrant was related to psychiatric problems (41.7%), including 2 with possible dementia (16.7%). However, 3 (25.0%) patients verbally insulted or physically assaulted staff members and other patients.

**CONCLUSION:**

Although the number of recalcitrant patients was small, we recommend that there should be one or two TB facilities readily available for law enforcement officials to enforce isolation.

In Japan, the TB notification rate has declined 85-fold in the past seven decades, from 698 per 100,000 population in 1951 to 8.2 in 2022.^1^ However, almost 4,000 smear-positive TB cases are still reported every year,^1^ with over 65% of the patients aged ≥65 years, reflecting the age shift of the infection pool from the young to the elderly. The Prevention of Infections and Medical Care for Patients of Infections law of 1998 stipulates that all infectious (often sputum smear-positive) patients with pulmonary TB must be isolated in designated hospitals with TB isolation beds to prevent these patients from spreading TB to the community.^2^ The patients are released from the TB beds once they have smear-negative sputum at least three consecutive times. Typically, once a physician reports an infectious patient with TB to the prefectural governor, the medical director of the local health office issues a recommendation letter to the patient instructing him or her to be isolated, and most patients oblige. During the isolation, all the medical costs related to anti-TB treatment, including some complications (e.g. hepatotoxicity and skin reaction) and surgery (if indicated), are covered by the national and local governments, the health insurance agency, or both.^3^

However, there has always been a small number of recalcitrant patients with TB who are difficult to isolate and who are mentally and/or physically abusive to healthcare workers and other patients in the ward.^4^ Although they cannot immediately be discharged from the hospitals because of fear of transmission of their TB to the community and the fear that, if they leave the hospital, they might not adhere to anti-TB treatment any longer. They may eventually self-discharge, be discharged despite the patient’s intention to be continuously hospitalised and treated, or be transferred from the original hospital to other hospitals, often to psychiatric hospitals, for various reasons. Such patients might be too depressed to remain isolated, abuse alcohol in the ward, insult or physically assault other patients or staff members, or commit theft.^4^

An earlier study reported that the proportion of recalcitrant patients with TB who were difficult to isolate from 2013 to 2014 was 0.5%.^4^ However, since almost 10 years have passed after the previous investigation, we conducted another survey of such recalcitrant patients with TB to characterise them in terms of time, place and person and to determine why they were self-discharged, discharged, or transferred.

## Study population, design and methods

A recalcitrant patient with TB who was difficult to isolate was defined as a patient with TB who initially was hospitalised in the TB isolation ward of a hospital and then either self-discharged, was discharged despite the patient’s intention to be continuously hospitalised and treated, or transferred to another hospital, mainly a hospital with a psychiatric ward, because of being unmanageable. Those patients with TB who were transferred due to medical and physical conditions, such as the need for intensive or cardiac care or haemodialysis, were excluded from the definition.

From July to October 2023, we sent a self-administered questionnaire by postal mail to the nursing directors of hospitals with TB isolation beds in Japan. The list of hospitals with TB isolation beds was obtained from Japan’s Ministry of Health, Labour and Welfare website.^5^ Considering the relative rarity of recalcitrant patients with TB, we limited the scope of hospitals to ones with five or more TB isolation beds. The questionnaire asked whether the hospital had experienced any recalcitrant inpatients with TB from April 2022 through March 2023, and, if so, we further inquired about the patients’ demographic information, the reasons why they left the hospital and how they were subsequently followed up. We also asked about the time period in which the patients were hospitalised in the ward. When multiple reasons were given, we chose the most serious and/or root cause reason as the main reason. For example, when both mental and physical assaults were reported, physical assaults were chosen as the main reason because of their seriousness. When physical and alcohol abuse were reported, alcohol abuse was chosen since it seemed to be the root cause of the difficulty in isolating the patient. When psychiatric problems were reported as the reasons for leaving the hospitals or transferring to another institution, they were taken at face value.

The number of sputum smear-positive TB patients registered from April 2022 through March 2023 was retrieved from the official surveillance data.^6^ We double-entered the data in an Excel 2010 (Microsoft Corp, Seattle, WA, USA) spreadsheet and compared them, and inconsistencies were resolved by returning to the original questionnaire.

The authors obtained approval from the institutional review board of the Research Institute of Tuberculosis (#2023-16), Tokyo, Japan.

## RESULTS

We sent questionnaires to 137 hospitals with five or more TB beds designated by the prefectures. Of these, 60 (43.8%) responded. Of the 60, 8 (13.3%) hospitals had 100 or more patients with TB during the study period, 9 (15.0%) between 60 and 99, 16 (26.7%) between 40 and 59, 10 (16.7%) between 20 and 39, and 17 (28.3%) less than 20. Eleven hospitals (18.3%) had patients with multidrug-resistant TB (MDR-TB) in the study period. Seven hospitals (11.7%) had experienced difficulty in isolating one or more recalcitrant patients during the study period.

A total of 12 recalcitrant patients were reported, eight of whom (66.7%) self-discharged. Four (33.3%) were discharged by the hospital, and none (0%) were transferred. All (100%) were male. We did not collect information about MDR-TB in the recalcitrant patients. The 12 patients corresponded to 0.3% (95% confidence interval [CI] 0.17–0.57) of the 3,703 patients with smear-positive TB reported from April 2022 through March 2023 nationwide who were supposed to be isolated initially.

**FIGURE 1.**
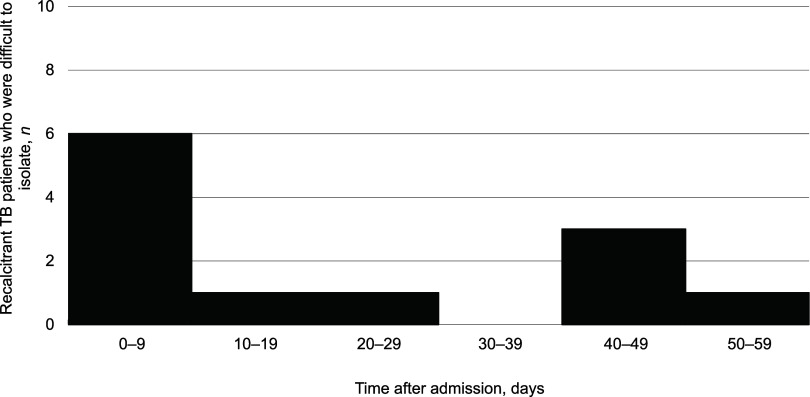
Onset of recalcitrance of patients with TB who were difficult to isolate, Japan, 2022–2023 (*n* = 12).

**FIGURE 2.**
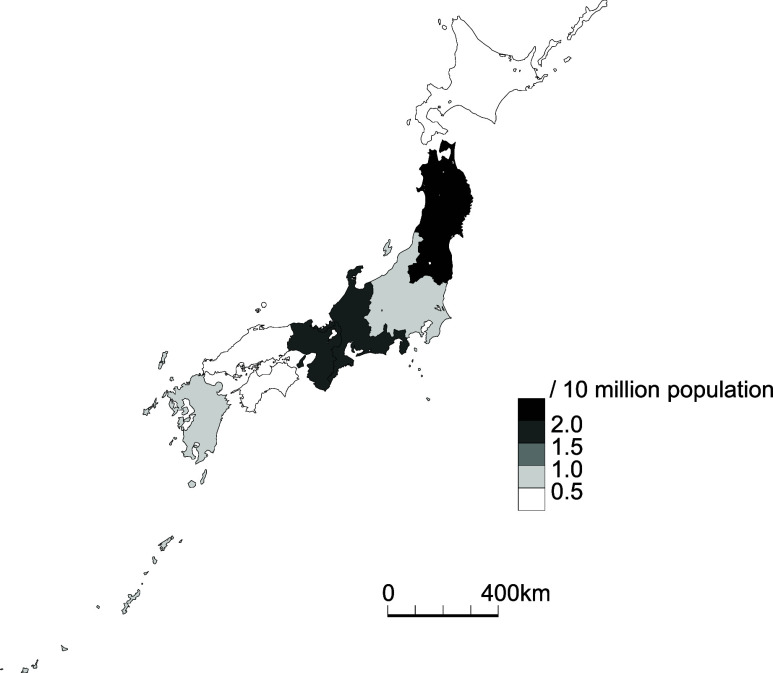
Geographic distribution of recalcitrant patients with TB who were difficult to isolate, Japan, 2022–2023.

**FIGURE 3.**
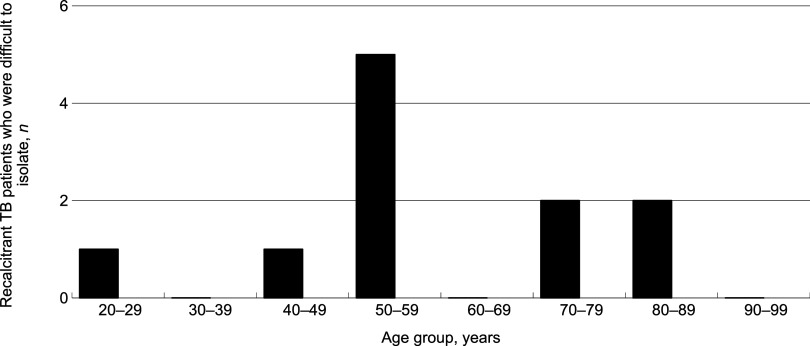
Age distribution of recalcitrant patients with TB who were difficult to isolate, Japan, 2022–2023 (*n* = 12).

Figure 1 shows the distribution of the timing of the onset of recalcitrance of TB patients who self-discharged or were discharged. There were two peaks, at 0–9 and 40–49 days after admission, during which these patients left the TB wards. Figure 2 shows the geographic distribution of rates of the recalcitrant patients with TB per 10 million population. The rate was highest in the Tohoku region (2 cases, 2.4/10 million, 95% CI 0.29–8.6), followed by the Tokai-Hokuriku (3 cases, 1.6/10 million, 95% CI 0.35–5.0) and the Kinki (3 cases, 1.5/10 million, 95% CI 0.3–4.3) regions. On the other hand, the Kanto-Kou-Shin-Etsu region, including the metropolitan Tokyo area, had the lowest rate (3 cases, 0.6/10 million, 95% CI 0.12–1.8), followed by the Kyushu-Okinawa region (1 case, 0.7/10 million, 95% CI 0.18–3.9). The Hokkaido and Chugoku-Shikoku regions both had no cases. There was no significant difference in the rates of recalcitrant patients with TB among the regions. Figure 3 shows the age distribution of the recalcitrant patients with TB. Their average age was 58.9 (95% CI 48.1–69.7) years (range: 21–80). Table 1 shows why they left the hospital (above) and how the TB patients were followed up after they left the original institutions (below). Five (41.7%) patients left the hospitals because of psychiatric problems, including two (16.7%) who seemed to have possible dementia, followed by assaults (*n* = 3, 25.0%), including two (*n* = 2, 16.7%) who committed physical assaults, on other patients and/or healthcare workers. Two (16.7%) patients strongly desired to go home and were not persuaded to return to the hospital. Two other (16.7%) patients were not able to stop smoking in the ward and left the hospital. In terms of follow-up of the recalcitrant patients with TB, one (8.3%) eventually returned to the isolation ward; another (8.3%) was seen at the outpatient department of the original hospital, and the other (83.3%) was followed up by the local health office staff.

**TABLE.** The reasons why the recalcitrant patients with TB left the hospital with TB isolation beds (A) and how they were followed up (B), Japan, 2022–2023.

**A**	
Reasons	n (%)
Psychiatric problems	5 (41.7)
Possible dementia	(2 (16.7))
Insults to other patients/HCWs	1 (8.3)
Physical assaults to other patients/HCWs	2 (16.7)
Strong desire to return home	2 (16.7)
Smoking	2 (16.7)
Subtotal	12 (100.0)
**B**	

How the patients were followed up	n (%)
Followed up by the local health office	10 (83.3)
Admission to the original hospital ward	1 (8.3)
Followed up at the outpatient clinic of the original hospital	1 (8.3)
Subtotal	12 (100.0)

HCW = healthcare workers

## DISCUSSION

We conducted a questionnaire survey on recalcitrant patients with infectious TB who were admitted to hospitals with TB beds in Japan from April 2022 through March 2023 but were difficult to isolate and eventually left the hospital ward. This was our second study, and we conducted a similar study in 2013–2014. Approximately one-ninth of the hospitals had such patients, who were all males. The proportion of the recalcitrant patients with TB among all the smear-positive patients registered in the same period was 0.3%. Approximately two-fifths of the reasons for their self-discharge or discharge were psychiatric problems, including possible dementia. However, it should be noted that a small number of the patients conducted assaults, including physical assaults, on the other patients and/or healthcare workers.

One of the reasons for the difficulty in isolating recalcitrant patients with TB was psychiatric problems. This might be because the period of isolation is often longer than one month, and life in the TB isolation ward may not be comfortable, mainly because the patients are restricted to the ward.^4,7^ Another reason may be that patients with TB might not be provided with psychosocial support from the hospital because of fear of psychiatrists and psychologists being infected with TB by the patients, and thus, the patients may experience depression during the isolation. Although it is a concern that psychiatrists and psychologists may be infected with TB when they see patients with smear-positive TB in their rooms, the likelihood of obtaining TB infection for the workers is negligible when the room is well-ventilated and/or they wear N95 masks. In general, patients with TB are becoming older and older in Japan^1,4,8–10^ because the TB infection pool is shifting to the elderly, and some of the elderly patients with TB may have dementia when they are hospitalised or have their dementia worsened due to the isolation environment. However, in the last decade, it has become much more difficult to accommodate patients having both infectious TB and psychiatric disorders, including dementia, as the number of psychiatrists who can also manage TB decreased dramatically. Some patients with TB who mentally or physically assaulted the hospital staff were forced to leave the hospitals because it was not possible to restrain their violent and/or abusive behaviour, and there were no high-security TB wards with law enforcement officials or security guards where such patients could physically be detained.

Compared with our previous study conducted in 2013, the proportion of recalcitrant patients among the patients with infectious TB decreased from 0.5% to 0.3%, a 36.0% reduction, although the difference was not statistically significant (*P* = 0.19). One of the reasons why the proportion may have decreased was that the average period of isolation for patients with infectious TB was shortened by 24.3 days (35.3%), from 68.8 days in 2013 to 44.5 days in 2022.^11,12^ The average period of isolation has gradually decreased in recent decades; however, the reduction in 2022 was partly due to the shortage of isolation beds in the hospitals caused by the COVID-19 pandemic.^13,14^ The reasons why the recalcitrant patients with TB left their hospitals were similar to those in the previous study, in which about two-fifths of the reasons were related to psychiatric problems and about one-fourth were insults or physical assaults on other patients and/or healthcare workers.^4^ However, one difference was related to smoking, which was not a reason in the previous study, though it was in the current study. Restrictions on cigarette use have become stricter in public spaces in Japan, including health care facilities, particularly in hospital environments, than in the past, which may have made the life of smokers with TB unbearable. A study conducted in a hospital in metropolitan Tokyo having the largest number of isolation beds reported that they had 76 recalcitrant patients with TB for the eleven-year-period from 1993 through 2003, of whom about one-fourth abused alcohol in the isolation ward and about one-seventh physically assaulted other patients and health care workers.^9^ The same study also reported that about one-seventh were transferred to psychiatric wards because of unmanageable mental and/or psychiatric problems. Even though the study was conducted about 20 years ago, the demographic distribution of patients with TB was quite different; although the study area was limited to metropolitan Tokyo, the situation was quite similar to that in the current study since a large portion (altogether about one-third) of the recalcitrant patients in the ward had psychiatric issues, including alcohol abuse.^15,16^ One of the interesting findings of our study was that none of the recalcitrant patients with TB reportedly used illicit drugs, unlike in other countries.^17–19^ This may be because illicit drug use is not as common in Japan (0.3% of the population were lifetime users in 2015) as in, for example, the United States (7.5% of the population were current users in 2013).

One of the strengths of this study is that the authors sent questionnaires about recalcitrant patients with TB to most of the hospitals with TB beds, and the findings are considered to be representative of the actual situation in Japan. However, our study also has limitations. First, the response rate of this study was not optimal, and our findings may have underestimated the actual number of such patients. On the other hand, because of the seriousness of the difficulty, we still believe that the hospitals that experienced that difficulty will respond to rather than ignore the questionnaire to be heard about the difficulty. Second, we did not send the questionnaire to hospitals with less than five TB beds, considering the small number of patients they hospitalise per year, and this may have caused another slight underestimation. Since this was a questionnaire survey, the authors were not able to verify the numbers of recalcitrant patients and why they were discharged, especially the patients’ psychological or behavioural issues. Finally, as this was a descriptive study, we did not perform analytic epidemiology to identify the factors associated with patients’ recalcitrance. Therefore, further research is required to clarify this issue.

We want to make several recommendations to decrease the number of recalcitrant patients with TB in the wards. First, the duration of hospital stay for patients with TB should be shortened as much as possible as most, though not all, patients with smear-positive TB may not spread TB to others and cause outbreaks.^20,21^ Currently, several polymerase chain reaction-based assays that can detect rifampicin and isoniazid resistance from sputum are commercially available,^22,23^ it is possible to exclude multidrug or rifampicin resistance on day 1 of admission. When such resistance is excluded, the duration of hospital stay can be shortened in appropriate cases. Second, as this and previous studies showed, the recalcitrant patients with TB left the hospital second most often at one month after the admission.^4^ Perhaps the Ministry of Health should consider home (or self) isolation for some of the patients who are cooperative and adherent to the infection prevention measures for TB and for whom continuity of treatment is assured. Third, TB hospitals should improve the environment for patients with TB, particularly in relation to amenities, including room space, walking space, a gym, a garden, an internet connection, and entertainment facilities in the TB ward or patient’s room so that the isolated patients with TB can live a more comfortable life in the isolation ward. Fourth, since the majority of recalcitrant patients have psychiatric problems, there should be a psychiatrist or a psychologist available to be consulted when the patients exhibit depression or psychiatric disorders. Fifth, since most patients with infectious TB are persuaded to be isolated and are not forcibly hospitalised if the patient does not agree to be isolated or disappears from the TB ward, law enforcement officials are not currently involved in the detention or pursuit of such patients. Because a small number of recalcitrant patients with TB, particularly young males, physically assault or insult other patients or TB hospital staff, there should be one or two TB facilities (presumably in Tokyo and Osaka) in Japan with law enforcement officials or security guards readily available to enforce isolation of recalcitrant patients with infectious TB, as is practised in Australia, Israel, the United States and several European countries.^24–28^
